# Retracted: Abnormal COX2 Protein Expression May Be Correlated with Poor Prognosis in Oral Cancer: A Meta-Analysis

**DOI:** 10.1155/2021/3284807

**Published:** 2021-02-12

**Authors:** BioMed Research International

*BioMed Research International* has retracted the article titled “Abnormal COX2 Protein Expression may be Correlated with Poor Prognosis in Oral Cancer: A Meta-Analysis” [1] due to the inclusion in the meta-analysis of an article cited as reference 22 that was retracted in 2007 for containing fabricated data [2].

In addition, this meta-analysis includes phrases characteristic of a series of very similar meta-analyses written by different authors that were published in 2014 and 2015 [3]. Egger's test was conducted with fewer than 10 studies, limiting its validity.

The authors and their institution did not respond.

## References

[1] Wang Z.-M., Liu J., Liu H.-B., Ye M., Zhang Y.-F., Yang D.-S. (2014). Abnormal COX2 Protein Expression May Be Correlated with Poor Prognosis in Oral Cancer: A Meta-Analysis. *BioMed Research International*.

[2] Sudbø J., Ristimäki A., Sondresen J. E. (2003). RETRACTED: Cyclooxygenase-2 (COX-2) expression in high-risk premalignant oral lesions. *Oral Oncology*.

[3] Filion G. (2014). *A flurry of copycats on PubMed*.

